# Repeated heat exposure upregulates skeletal muscle aquaporin‐4 expression in male mice

**DOI:** 10.14814/phy2.70926

**Published:** 2026-05-19

**Authors:** Yuka Kudo, Nozomi Yazawa, Yui Shimizu, Atsumi Tomatsuri, Yuho Mizuseki, Kazuko Koshiba‐Takeuchi, Taku Nedachi

**Affiliations:** ^1^ Department of Life Sciences, Graduate School of Life Sciences Toyo University Asaka Saitama Japan; ^2^ Faculty of Life Sciences Toyo University Asaka Saitama Japan

**Keywords:** aquaporins, heat acclimation, heat exposure, skeletal muscle

## Abstract

Heat acclimation involves coordinated physiological adaptations, yet the role of skeletal muscle water channels remains insufficiently defined. We investigated how repeated heat exposure affects aquaporin‐4 (AQP4) in skeletal muscle using both in vivo and in vitro models. Male C57BL/6NJ mice were exposed to daily passive heating (45°C for 30 min/day, 14 days). Repeated heat exposure reduced body‐weight gain and markedly attenuated *Hspa1a* induction following an acute heat challenge, confirming systemic heat acclimation. Immunofluorescence analysis revealed an approximately 1.3‐fold increase in AQP4 protein in fast‐twitch muscles without changes in its subcellular localization. In vitro, C2C12 myocytes were subjected to repeated heat bouts (42°C, 3 h/day for 1–5 days). Heat‐acclimated cells exhibited blunted *Hspa1a* induction in response to acute heat stress and progressively increased AQP4 protein levels (approximately 1.6‐fold), indicating that heat stress directly promotes AQP4 upregulation in muscle cells. Together, these findings identify AQP4 as a heat‐responsive component of skeletal muscle and suggest that AQP4 may contribute to peripheral osmotic regulation during heat acclimation.

## 1 INTRODUCTION

Increased environmental temperatures or internal heat production during exercise elicit a series of physiological responses in the body. These include enhanced sweating, increased skin blood flow, cardiovascular adjustments, and body‐fluid regulation, all of which act to dissipate excess heat and minimize thermal strain (Tansey & Johnson, 2015). Such coordinated adaptations have been well documented and can mitigate physiological perturbations while maintaining physical performance under thermal stress (Périard et al., 2021). However, excessive and/or prolonged heat exposure can lead to heat‐related illnesses, including life‐threatening emergencies such as heatstroke, which may cause permanent damage to the brain, heart, kidneys, muscles, and other organs (Epstein & Yanovich, 2019; Yoneda et al., 2024).

Heat acclimation, which is the physiological adaptation to repeated heat exposure, has gained increasing attention as a preventive measure against heat‐related illness. When mild heat stress exposure is repeatedly applied, heat acclimation (HA) typically develops within 7–14 days (Tyler et al., 2016). This process induces increased sweating, improved cutaneous vasodilation, decreased cardiovascular strain, and improved exercise performance (Shaw et al., 2022; Tyler et al., 2016). Among these adaptations, plasma volume expansion has consistently been identified as a central feature, contributing to more efficient heat dissipation and stabilization of circulatory function (Oberholzer et al., 2019; Périard et al., 2021; Senay Jr., 1972).

Aquaporins (AQPs), a family of membrane channel proteins, contribute to rapid and selective water transport across biological membranes (Agre et al., 2002). At least 13 mammalian isoforms (AQP0–AQP12) have been identified, each showing distinct tissue distributions and physiological functions (Agre et al., 2002). Several AQPs (e.g., AQP1, AQP2, and AQP4), which are classified as orthodox aquaporins, mainly transport water. For instance, AQP1 in endothelial cells facilitates capillary water permeability (Verkman & Mitra, 2000), AQP2 in renal collecting ducts regulates urinary concentration under vasopressin control (Rojek et al., 2006), and AQP4 in astrocytes contributes to brain water balance and edema formation (Papadopoulos & Verkman, 2007). These isoform‐specific distributions and regulatory mechanisms imply that AQPs act as key molecular determinants of water homeostasis under physiological and stress conditions.

From the perspective of body‐fluid homeostasis, skeletal muscle serves as a large water reservoir and acts as a dynamic buffer contributing to body‐fluid redistribution during thermal stress (Hackney et al., 2012; Trangmar & González‐Alonso, 2019). In the skeletal muscle, the water channel protein AQP4 primarily facilitates transmembrane water transport (Frigeri et al., 1998). AQP4 is preferentially expressed in fast‐twitch fibers, localized to the sarcolemma, and contributes to osmotic balance and cell volume regulation as observed from histological studies (Frigeri et al., 1998; Wakayama, 2010). Furthermore, endurance or resistance exercise upregulates AQP4 expression, whereas genetic deletion of AQP4 impairs osmotic recovery and changes the contractile properties (Basco et al., 2011, 2013). These results suggest that environmental stresses, including those linked to heat exposure, could affect muscle AQP4 expression. However, direct evidence that heat stress per se modifies AQP4 expression in skeletal muscle remains scarce.

In other tissues, heat exposure changes the expression of aquaporin family members (Aloui et al., 2024; Sugimoto et al., 2013; Wang et al., 2015). Studies using rodent and avian models show that thermal stress influences AQP1, AQP2, and AQP4 in the hypothalamus, kidney, and intestine, suggesting that aquaporin systems show heat sensitivity (Aloui et al., 2024; Sugito et al., 2021; Wang et al., 2015). These results imply that the AQPs contribute to systemic water homeostasis during thermal adaptation.

Together, these results underscore the physiological criticality of body‐fluid regulation during heat acclimation. However, the role of skeletal muscle AQP4 in thermal adaptation remains largely unexplored. Given that the skeletal muscle constitutes a large water reservoir and undergoes substantial fluid shifts during heat stress as described, alterations in muscle AQP4 expression could affect local muscle function and systemic fluid distribution during heat acclimation. Clarifying whether repeated heat exposure modulates AQP4 expression and localization within skeletal muscle could provide new insights into the peripheral mechanisms that support body‐water regulation during heat acclimation. Herein, we aimed to assess the effects of repeated heat exposure on AQP4 expression and distribution in skeletal muscle, under in vivo and in vitro conditions.

## 2 MATERIALS AND METHODS

### 2.1 Animals and heat exposure

The Animal Care Committee at Toyo University approved all animal experiments. Male C57BL/6NJ mice (The Jackson Laboratories Japan Inc., Kanagawa, Japan; 7 weeks of age at the beginning of the experimental protocol) were individually housed in a temperature‐ and humidity‐controlled room. The animals were fed chow (Labo MR Stock, Nosan Corp., Kanagawa, Japan) and provided water ad libitum and subsequently acclimatized to a 12‐h light cycle (lights on between 0800 and 2000 h) for 1 week before experimental manipulation. During this week, the mice were assigned to individual cages. Subsequently, mice were randomly assigned to control or heat groups. Heat exposure was delivered in a calibrated environmental chamber (Nippon Medical & Chemical Instruments Co., Ltd., Osaka, Japan) at 45°C for 30 min/day for 14 consecutive days. The temperature and duration were selected based on our previous findings demonstrating that a single exposure to 45°C induces heat shock protein expression in skeletal muscle (Murata et al., 2023). This approach was further supported by prior studies employing acute heat stress in rodents (Audet et al., 2017; Welc et al., 2012). The final conditions were optimized in preliminary experiments to ensure sufficient induction of heat shock responses without causing overt tissue damage. On the experimental day (Day 15), control mice and heat‐treated mice (exposed to 45°C for 45 min) were anesthetized and were immediately euthanized by cervical dislocation in accordance with the institutional guidelines, and the tibialis anterior (TA), extensor digitorum longus (EDL), quadriceps (Quad), and soleus (SOL) muscles were removed, frozen in liquid nitrogen (EDL: 30 s, Quad: 1 min), and then stored at −80°C.

### 2.2 Cell culture

The mouse skeletal muscle cell line C2C12 was maintained as previously described (Ishiuchi et al., 2018). C2C12 myoblasts were cultured in Dulbecco's modified Eagle's medium (DMEM) supplemented with 10% fetal bovine serum (S1400, BioWest, Nuaille, France), 30 μg/mL penicillin, and 100 μg/mL streptomycin at 37°C in a 5% CO_2_ atmosphere. For all experiments, cells were grown in a 35‐mm dish (631‐33121, AGC Techno Glass Co., Ltd., Shizuoka, Japan) at a density of 2.0 × 10^5^ cells/well in 2 mL growth medium. Cell differentiation was induced by incubating them in differentiation medium (DMEM supplemented with 2% calf serum (CS; S0400, BioWest), 30 μg/mL penicillin, and 100 μg/mL streptomycin) for 6 days when the cells reached 100% confluence 2 days after plating. The medium was changed every 24 h after inducing differentiation. Repeated heat exposure (42°C, 3 h/day for 1–5 days) was applied in a CO_2_ incubator. On the final day, with or without a 3 h heat bout, cells were immediately harvested for RNA and protein extraction.

### 2.3 Reverse transcription‐quantitative polymerase chain reaction (qPCR)

Total RNA was isolated using TRIzol™ reagent (Thermo Fisher Scientific Inc., Waltham, MA, USA) in accordance with the manufacturer's instructions. Total RNA concentration was quantified using NanoDrop™ 2000/2000c (Thermo Fisher Scientific Inc.). Reverse transcription was performed using PrimeScript™ RT Master Mix (Perfect Real Time) (RR036, Takara Bio, Shiga, Japan), and the obtained cDNA was diluted 10‐fold with EASY Dilution (for real‐time PCR; Takara Bio). Subsequently, qPCR was conducted using the QuantStudio One™ real‐time PCR system (Applied Biosystems, Waltham, MA, USA) and Thunderbird® SYBR qPCR Mix (QPS‐201, Toyobo Co., Ltd., Osaka, Japan). All samples were analyzed in technical triplicates. The sequences of the primers used were mouse *Hspa1a*, 5′‐TGGTGCAGTCCGACATGAAG‐3′ and 5′‐GCTGAGAGTCGTTGAAGTAGGC‐3′; mouse *Aqp4*, 5′‐CTGGAGCCAGCATGAATCCAG‐3′ and 5′‐TTCTTCTCTTCTCCACGGTCA‐3′; and mouse *Gapdh*, 5′‐TGTGTCCGTCGTGGATCTGA‐3′ and 5′‐CGTGCTTCACCACCTTCTTGA‐3′.

### 2.4 Western blotting

Cells were harvested using a cell extract solution [50 mM Tris–HCl (pH 7.6), 150 mM NaCl, 1 mM EDTA, 1% TritonX‐100, 0.1% Protease Inhibitor Cocktail (25955‐11, Nacalai Tesque, Kyoto, Japan)] and subsequently centrifuged at 4°C, 13,200 × *g* for 10 min. The supernatant was added with 2 × sample buffer [0.1 M Tris–HCl (pH 6.8), 4% sodium dodecyl sulfate (SDS), 20% glycerol, 0.01% bromophenol blue], and 1% 2‐mercaptoethanol. The cells were resolved by 12% SDS‐polyacrylamide gel electrophoresis. Proteins were transferred onto polyvinylidene difluoride membranes (IPVH00010, MERCK Millipore, Billerica, MA, USA). The membranes were blocked with 3% bovine serum albumin (01859‐47, Nacalai Tesque) in TBS‐T at room temperature for 30 min and subsequently incubated at 4°C overnight with primary antibodies, including anti‐HSP70 antibody (10995‐1‐AP, Proteintech, Rosemont, IL, USA, 1:10000), anti‐AQP4 antibody (#59678S, Cell Signaling Technology, Danvers, MA, USA, 1:1000), and anti‐β‐actin antibody (60008‐1‐lg, Cell Signaling Technology, 1:1000). The membranes were washed three times with TBS‐T for 10 min and incubated with a secondary antibody [horseradish peroxidase‐linked anti‐mouse or anti‐rabbit IgG (7076S or 7074S, Cell Signaling Technology, 1:5000)] at room temperature for 1 h. The signal for each protein was visualized using enhanced chemiluminescence detection (02230‐30, Nacalai Tesque) and iBright CL750 Imaging System (Thermo Fisher Scientific Inc.). Each band was quantified using ImageJ 1.54g (https://imagej.nih.gov/ij/).

### 2.5 Immunofluorescence for mouse skeletal muscle

For immunostaining, serial cross‐sections (10 μm thick) were cut using a cryostat at −22°C to −24°C, thawed on MAS‐coated slide glass (Matsunami, Osaka, Japan), and stored at −80°C. Samples were pre‐warmed at 37°C for 30 min before the experiment, and subsequently treated with 4% paraformaldehyde/PBS for 15 min at room temperature. The samples were washed with PBS‐T, blocked in PBS containing 5% BSA and 0.1% Triton X‐100 for 30 min, and incubated with the primary antibody against AQP4 (#59678S, Cell Signaling, 1:200) at 4°C overnight. The samples were washed three times with PBS‐T for 5 min and subsequently incubated with a secondary antibody [Alexa Fluor 488 Goat Anti‐Rabbit IgG H&L (ab150077, Abcam, Cambridge, UK, 1:200)] at room temperature for 1 h. After incubation with the secondary antibody, the cells were washed three times with PBS‐T for 5 min each, stained with DAPI (Sigma‐Aldrich Japan, Tokyo, Japan, 5 μg/mL) for 15 min, and mounted with VECTASHIELD mounting medium (H‐1000, Vector Laboratories, Burlingame, CA, USA). Fluorescence images were acquired using a confocal fluorescence microscope (CSU‐WI/IX‐83P2ZF/FV31‐SPC0V, Olympus, Tokyo, Japan) equipped with CellSens Dimension 2.3 (Olympus). For each muscle from each mouse, one section was analyzed, and 50 fields were imaged per section. Quantification was performed across fields, and values are presented to reflect the distribution of signal intensity.

### 2.6 Statistical analysis

All experiments were repeated at least three times. Statistical analyses were conducted using GraphPad Prism 6.0a (GraphPad Software, CA, USA). Student's *t*‐test was used for two‐group comparisons. Comparisons among treatment groups were performed using one‐way analysis of variance (ANOVA) with Tukey's post‐test or Student's *t*‐test. When two independent variables were involved, two‐way ANOVA was used to evaluate the main effects and their interaction. Differences were considered statistically significant at *p* < 0.05.

## 3 RESULTS

### 3.1 Repeated heat exposure attenuates body weight gain and Hspa1a induction in mouse skeletal muscle

**FIGURE 1:**
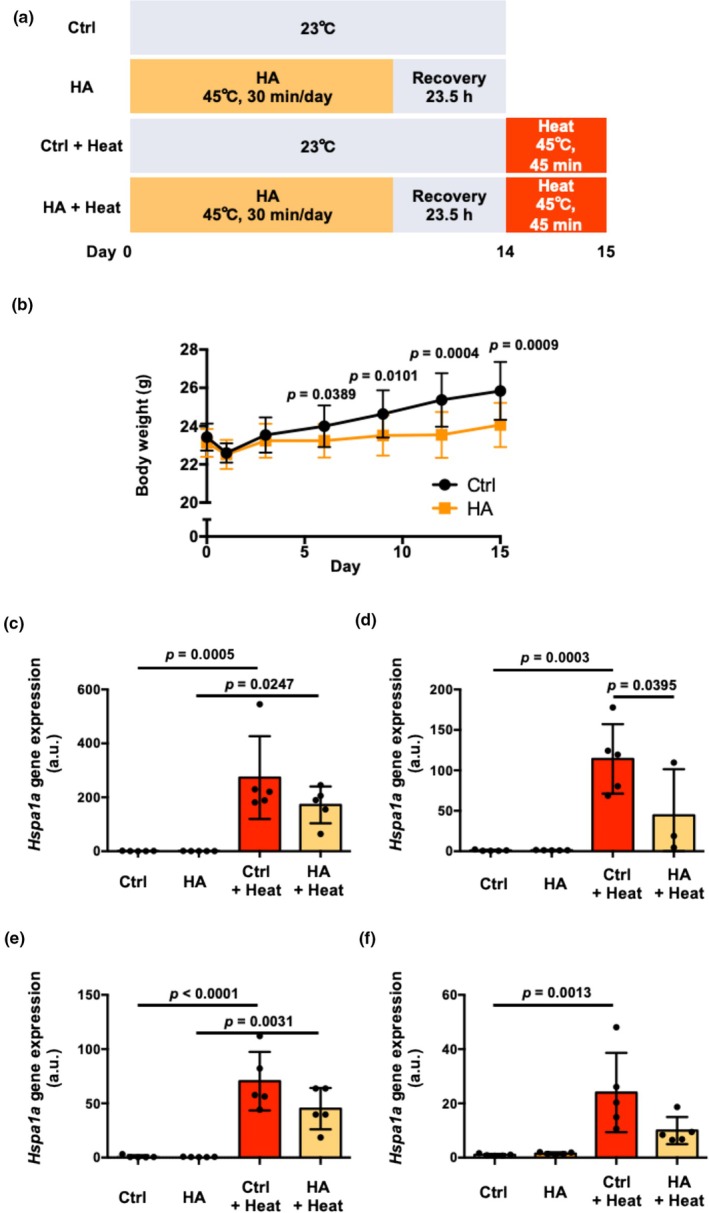
Repeated heat exposure suppressed both body weight gain and skeletal muscle heat responsiveness. (a) Experimental schedule: Male C57BL/6NJ mice aged 7 weeks were exposed to 45°C heat stress for 30 min daily over 14 days. (b) The body weight of each group was measured every three days. The graph represents the mean ± standard deviation (SD), and the data were analyzed using a one‐way analysis of variance (ANOVA) followed by Tukey's multiple comparison test (*n* = 5–17). Exact *p* values are shown in the figure. (c–f) On the experimental day, control mice, heat‐treated mice (exposed to 45°C for 45 min), were anesthetized, and tibialis anterior (TA), extensor digitorum longus (EDL), quadriceps (Quad), and soleus (SOL) muscles were removed as described in the Materials and Methods section. *Hspa1a's* relative expression in TA (c), EDL (d), Quad (e), and SOL (f) was analyzed using quantitative PCR. The graph represents the mean ± SD, and the data were analyzed using a one‐way ANOVA followed by Tukey's multiple comparison test (*n* = 3–5). Exact *p* values are shown in the figure.

Male C57BL/6NJ mice aged 7 weeks were exposed to 45°C heat stress for 30 min daily over 14 days (experimental schedule shown in Figure 1a). The body weight of each mouse was measured every 3 days. The body weight of mice repeatedly exposed to heat stress significantly decreased by day 6 and remained lower at day 15 compared with the control group (Figure 1b; control: 25.8 ± 1.5 g; repeated heat exposure: 24.1 ± 1.2 g; *p* = 0.0009, *n* = 5–17). Thus, repeated heat exposure negatively affected body weight gain in mice.

On the final day, an acute heat challenge (45°C for 45 min) was applied, and skeletal muscle samples, tibialis anterior (TA), extensor digitorum longus (EDL), quadriceps (Quad), and soleus (SOL), were immediately collected for RNA extraction and quantitative analysis of *Hspa1a* mRNA expression. All tested skeletal muscles in the repeated heat group demonstrated a decreased *Hspa1a* induction (TA: 0.63‐fold; EDL: 0.39‐fold; Quad: 0.64‐fold; SOL: 0.42‐fold; *n* = 3–5) compared with the controls (Figure 1c–f). Thus, repeated heat exposure suppressed body weight gain and skeletal muscle heat responsiveness, implying heat acclimation development.

### 3.2 Alterations in muscle hydration and AQP4 expression under repeated heat stress

**FIGURE 2:**
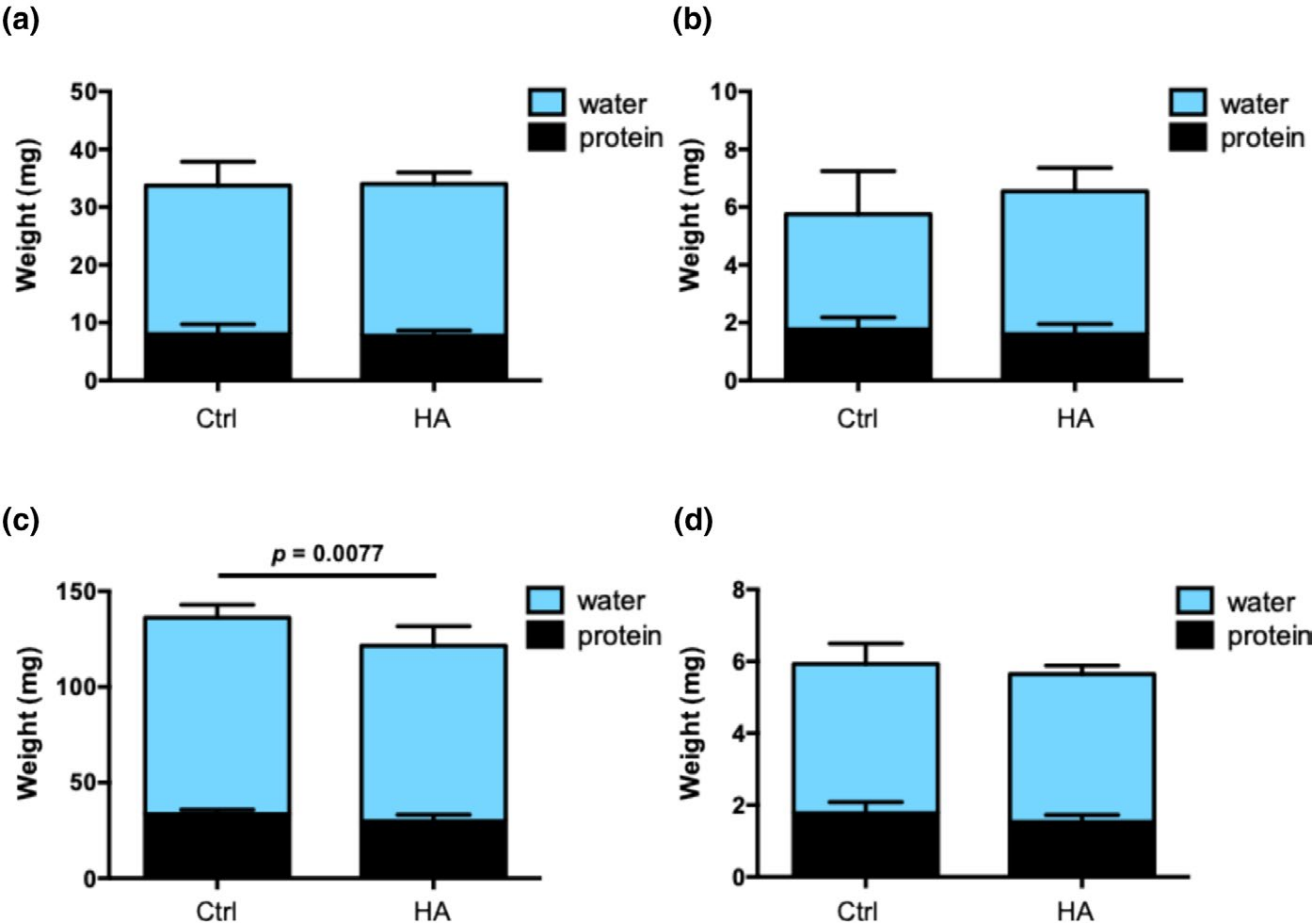
Effects of repeated heat exposure on water content in mouse skeletal muscle. (a–d) Male C57BL/6NJ mice aged 7 weeks were exposed to 45°C heat stress for 30 min daily over 14 days. Wet weight of each dissected muscle was measured immediately following excision. Dry weight was determined after oven‐drying the muscles at 100°C for 24 h until a constant mass was achieved, and water content was calculated as the difference between wet and dry weights (a: TA, b: EDL, c: Quad, and d: SOL). The graph represents the mean ± SD, and the data were analyzed using a two‐way ANOVA (*n* = 7). Exact *p* values are shown in the figure.

We next measured the wet and dry weights of TA, EDL, Quad, and SOL muscles to assess whether repeated heat exposure affected muscle hydration status (Figure 2). Dry weight was determined following oven‐drying the dissected muscles at 100°C for 24 h until a constant mass was achieved. Additionally, water content was calculated as the difference between wet and dry weights. No significant change was noted in the dry weight of either muscle; however, the water content of the Quad muscle was slightly but significantly reduced in the repeated heat group compared with the control (Figure 2c; control: 102.7 ± 6.8 mg; heat: 91.7 ± 10.1 mg; *p* = 0.0077, *n* = 7). Therefore, repeated heat exposure induces a mild decrease in skeletal muscle water content without affecting overall tissue mass, which may be consistent with mild dehydration and/or altered osmotic regulation during heat acclimation.

**FIGURE 3:**
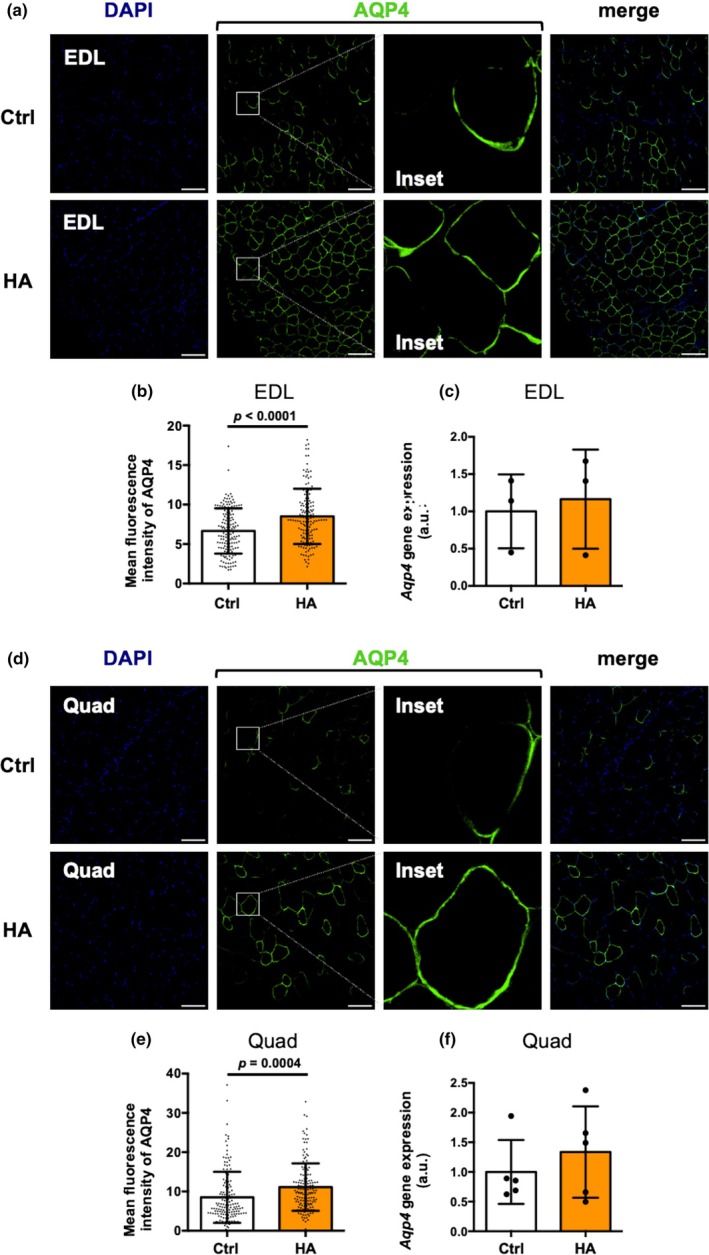
Repeated heat exposure improved AQP4 protein expression in the Quad and EDL muscles. (a–f) Male C57BL/6NJ mice aged 7 weeks were exposed to 45°C heat stress for 30 min daily over 14 days, and EDL (a–c) and Quad (d–f) muscles were obtained. (a, d) Immunofluorescent analysis using anti‐AQP4 antibodies was conducted as described in the Materials and Methods section. (b, e) Signal intensity obtained from (a) and (d) was measured using ImageJ. At least three independent experiments were conducted, and at least 50 spots were randomly selected from each experiment. The graph represents the mean ± SD, and the data were analyzed using a Student's *t*‐test (*n* = 150). Exact *p* values are shown in the figure. (c, f) Quantitative polymerase chain reaction analysis was performed to measure the *Aqp4* gene expression as described in the Materials and Methods section (not significant; *n* = 3–5).

As previously described, AQP4 is predominantly expressed in fast‐twitch muscles. Thus, we assessed AQP4 protein expression in the EDL (Figure 3a,b) and Quad (Figure 3d,e) muscle of the control and repeated heat groups via immunofluorescence staining (Figure 3a,d), followed by quantitative analysis using ImageJ (Figure 3b,e). Repeated heat exposure significantly increased AQP4 protein expression in EDL and Quad muscle by approximately 1.3‐fold (Figure 3b,e; *p* < 0.0001 and *p* = 0.0004, respectively, *n* = 150). However, no major differences were noted in the intracellular localization pattern of AQP4. Furthermore, qPCR analysis showed no significant alteration in the *AQP4* mRNA levels between the groups (Figure 3c,f; *n* = 3–5). This suggests that the increased AQP4 protein expression was post‐transcriptionally regulated. Collectively, these findings show that repeated heat exposure improves AQP4 protein expression at least in fast‐twitch skeletal muscle.

### 3.3 Repeated heat exposure induces cellular thermotolerance and AQP4 upregulation in C2C12 myocytes

**FIGURE 4:**
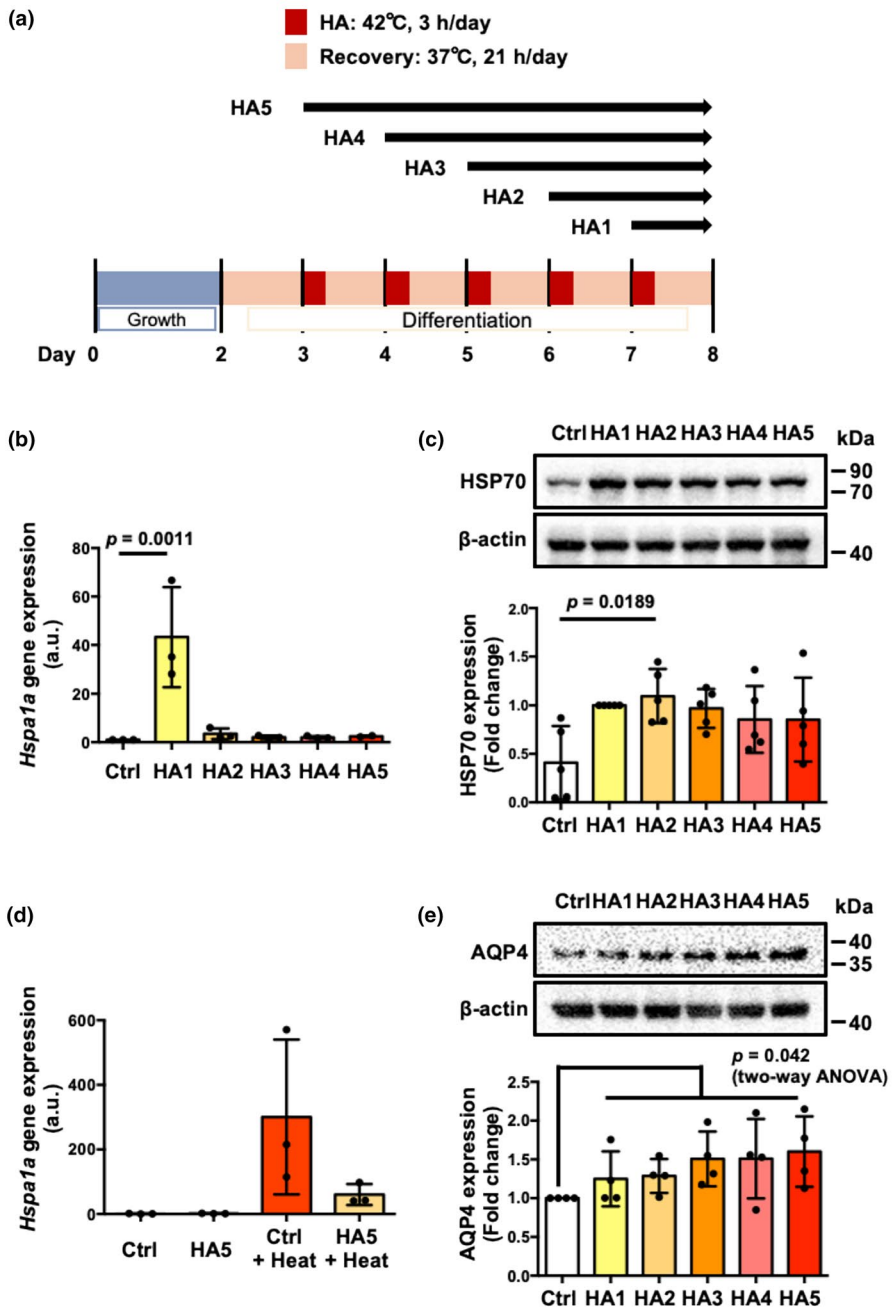
Repeated heat exposure improved AQP4 protein expression in the C2C12 myocytes. (a) Experimental schedule: C2C12 myoblasts were differentiated into myotubes as described and were exposed to 42°C heat stress for 3 h daily for 5 days. (b–e) On the final day, 21 h following the last heat exposure, total RNA and proteins from C2C12 myotubes were collected for analyzing *Hspa1a* gene expression (b) and HSP70 protein expression (c). Because the HSP70 signal in the Ctrl group was extremely low and close to the detection limit, HSP70 protein levels in (c) were normalized to HA1. (d) On the final day, C2C12 myotubes were exposed to 42°C heat stress for 3 h, after which total RNA was collected to analyze *Hspa1a* gene expression as described in the Materials and Methods section. (e) AQP4 protein expression was monitored using Western blotting analysis (two‐way ANOVA, main effect of heat acclimation, *n* = 4). Full‐length blots with molecular weight markers are provided in Figure S1. All graphs represent the mean ± SD obtained from these experiments (*n* = 2–5). Exact *p* values are shown in the figure.

We conducted in vitro experiments using C2C12 myocytes to determine whether the heat‐induced increase in AQP4 expression was a direct effect on skeletal muscle. During the differentiation process, C2C12 myocytes were subjected to repeated heat exposure at 42°C for 3 h per day for 1–5 days (experimental schedule in Figure 4a). On the final day, 21 h after a final heat exposure at 42°C, cells were collected for RNA and protein extraction. Analysis of *Hspa1a* mRNA expression revealed that in the single bout heated group (HA1), *Hspa1a* expression increased approximately 40‐fold, whereas in cells previously exposed to heat (HA2–HA5), *Hspa1a* induction was markedly attenuated (Figure 4b; *n* = 2–3). Contrastingly, the HSP70 protein remained evident even 21 h following heat exposure that had experienced heat (HA1–HA5), and HSP70 expression tended to be suppressed in proportion to the number of previous heat exposures (Figure 4c; *n* = 5). Additionally, *Hspa1a* induction tended to be suppressed in heat‐acclimated cells (Figure 4d; not significant, *n* = 3) when the cells were subjected to an additional 3 h heat exposure on the final day, demonstrating cellular thermotolerance.

Next, we assessed AQP4 protein expression. Western blot analysis revealed that heat acclimation significantly increased AQP4 expression compared with control (Figure 4e; two‐way ANOVA, main effect of heat acclimation, *p* = 0.042, *n* = 4), whereas no significant day‐dependent effect was detected across 1–5 days of acclimation. Although the day factor did not reach statistical significance, AQP4 expression showed an increasing trend with repeated heat exposure, with cells exposed to five cycles of heat stress showing an approximately 1.6‐fold increase in AQP4 protein expression compared with unheated controls.

Taken together, these findings indicate that *Hspa1a* induction attenuation and AQP4 expression upregulation noted in mice after repeated heat exposure are likely owing to direct effects of heat stress on the skeletal muscle cells, reflecting cellular mechanisms underlying heat acclimation.

## 4 DISCUSSION

### 4.1 Validation of the heat acclimation models and HSP70 responses

Herein, we utilized repeated passive heat exposure in mice (45°C, 30 min/day for 14 days) and C2C12 myocytes (42°C, 3 h/day for 1–5 days) to model systemic and cellular heat acclimation (Figures 1a and 4a). Classical rodent models of heat acclimation often use continuous or prolonged hyperthermia for several days and have reported attenuated rises in core temperature and blunted heat shock responses during subsequent heat challenges (Maloyan et al., 1999; Sareh et al., 2011). Similarly, human studies show that repeated exercise or passive heating in the heat modifies HSP70 responses to acute heat (McClung et al., 2008). The combination of decreased body‐weight gain and attenuated *Hspa1a* induction in our mice (Figure 1), together with the diminished HSP70 response in C2C12 cells (Figure 4), is therefore consistent with the acquisition of systemic and cellular heat acclimation rather than a simple acute stress response. However, because food and water intake were not monitored in the present study, it remains unclear whether the reduction in body‐weight gain reflects heat‐induced adaptation per se or secondary changes in energy intake and/or hydration status. Although our protocol used relatively short daily heat bouts, the direction of the observed adaptations closely resembles that reported in established models and supports our approach's validity.

HSP70, including the inducible 72‐kDa isoform (HSP72), is widely recognized as a key molecular marker and effector of heat acclimation (Amorim et al., 2015; Maloyan et al., 1999). Meta‐analyses and experimental studies in humans have revealed that repeated heat exposure changed the magnitude and kinetics of HSP70 induction upon subsequent heat stress (Nava & Zuhl, 2020). Similar phenomena have been reported in rodent organs, where heat‐acclimated animals attenuated HSP72 increases during acute heat challenges (Nava & Zuhl, 2020). Our observation that *Hspa1a* induction is blunted in multiple skeletal muscles following repeated heat exposure in in vivo and in vitro settings (Figures 1b–f and 4b–d) fits well with the concept of cellular thermotolerance, in which previous heat exposure decreases the requirement for prominent acute HSP surges.

### 4.2 AQP4 regulation under repeated heat stress in mouse skeletal muscle

Given that dehydration impairs cardiovascular stability, thermoregulation, and exercise performance, maintenance of body water and plasma volume is considered a central heat acclimation component (Millard‐Stafford et al., 2024; Sawka & Montain, 2000). Skeletal muscle, which contains approximately 75% water by mass (Costill et al., 1976; Fitts, 2008), is a major reservoir that can contribute to whole‐body water shifts during heat stress (Millard‐Stafford et al., 2024). In our model, repeated heat exposure did not alter muscle dry mass but moderately decreased water content in Quad muscle, suggesting subtle changes in muscle hydration without overt muscle wasting (Figure 2). The direction of this change might reflect chronic low‐grade dehydration, osmotic adjustments at the tissue level, or water redistribution between compartments. The functional relevance of this local muscle dehydration remains uncertain, considering that the magnitude of change was modest. However, AQP4 upregulation by repeated heat stimulation (Figure 3) may suggest that increased water channel availability may be associated with adaptation to osmotic fluctuations during repeated heat stress.

Aquaporins are increasingly recognized as crucial water balance regulators during heat and dehydration stress. For instance, in rat salivary glands, 5 days of continuous heat exposure upregulated AQP1 and AQP5 expression and improved apical AQP5 localization, which was proposed to contribute to evaporative cooling via increased salivary secretion (Sugimoto et al., 2013; Wang et al., 2009). Additionally, heat stroke and heat stress modulate aquaporin expression in the intestine and kidney, potentially contributing to changed fluid absorption, barrier integrity, and acid–base balance (Ariyo et al., 2024; McClung et al., 2008). Conversely, mild heat stress can downregulate AQP4 while upregulating AQP1, AQP2, and AQP5 in cultured fibroblasts, underscoring tissue‐specific regulation (Sugimoto et al., 2012). Against this background, our finding that repeated heat exposure increases AQP4 protein in fast‐twitch skeletal muscle adds a novel example of aquaporin‐mediated adaptation to thermal stress.

### 4.3 AQP4 in fast‐twitch muscle as a target of heat acclimation

AQP4 is highly enriched in the sarcolemma of fast‐twitch fibers and contributes to the high apparent water permeability of these fibers relative to slow‐twitch muscle (Frigeri et al., 1998). Multiple pathological conditions, including muscular dystrophies and denervation, are linked to decreased AQP4 expression, and such reductions have been implicated in impaired muscle function and atrophy (Crosbie et al., 2002; Ishido & Yoshikado, 2021). These observations generally support the view that AQP4 is beneficial for skeletal muscle homeostasis. In our study, repeated heat exposure increased AQP4 protein content in EDL and Quad muscle without changing its subcellular localization (Figure 3). This implies that heat acclimation may increase AQP4 abundance at the existing sarcolemmal sites rather than inducing ectopic redistribution. In the present study, *Aqp4* mRNA levels did not show a statistically significant change following repeated heat exposure. However, this result should be interpreted with caution, as the relatively small sample size and variability in the data may have limited the statistical power to detect subtle changes. Therefore, the absence of statistical significance does not necessarily exclude a modest transcriptional response of *Aqp4* to heat exposure.

Our C2C12 experiments show that repeated heat exposure directly increases AQP4 protein abundance in the myocytes. AQP4 in skeletal muscle can be regulated by innervation state and ubiquitin–proteasome pathways; for instance, Atrogin1‐mediated ubiquitination promotes AQP4 degradation and contributes to muscle atrophy (Chung et al., 2020). Therefore, repeated heat stress may suppress AQP4 degradation or improve its insertion into the sarcolemma, possibly via signaling pathways activated by heat, including HSP70‐dependent chaperoning, mitogen‐activated protein kinases, Ca^2+^‐dependent channels such as TRPV4, or reactive oxygen species (Caterina et al., 1997; Güler et al., 2002; Kawasaki et al., 1996; Li et al., 2017). Although our data do not identify the mechanism(s), the combined in vivo and in vitro results support a model in which cellular heat acclimation involves reprogramming of the heat shock response and remodeling of membrane water transport capacity via AQP4.

## 5 CONCLUSION

In conclusion, repeated heat exposure in mice and C2C12 myocytes induces a coordinated set of adaptations characteristic of heat acclimation, comprising attenuated *Hspa1a*/HSP70 responses to acute heat and increased AQP4 protein expression in fast‐twitch skeletal muscle. However, several limitations of the present study should be acknowledged. This includes potential sex‐, age‐, and strain‐dependent differences in heat acclimation and HSP responses. Importantly, the present study does not directly assess functional outcomes such as water transport capacity, osmotic recovery, or muscle performance. Therefore, the physiological significance of AQP4 upregulation remains to be determined. Future studies incorporating functional assays will be required to clarify the role of AQP4 in heat‐induced adaptations. Nevertheless, our findings support the validity of our mouse and cell paradigms as experimental models of heat acclimation and suggest that skeletal muscle may represent an important site associated with water handling during heat acclimation.

## AUTHOR CONTRIBUTIONS

**Yuka Kudo:** Conceptualization; data curation; formal analysis; investigation; visualization. **Nozomi Yazawa:** Formal analysis; investigation. **Yui Shimizu:** Formal analysis; investigation. **Atsumi Tomatsuri:** Formal analysis; investigation. **Yuho Mizuseki:** Formal analysis; investigation. **Kazuko Koshiba‐Takeuchi:** Supervision; validation. **Taku Nedachi:** Conceptualization; funding acquisition; methodology; project administration; supervision; validation.

## FUNDING INFORMATION

A Grant‐in‐Aid for Scientific Research from the Japan Society for the Promotion of Science (KAKENHI, C‐ 25K14942 to TN) supported this work.

## CONFLICT OF INTEREST STATEMENT

None.

## ETHICS STATEMENT

The animal experiments in this study were approved by the Institutional Animal Care and Use Committee of Toyo University and were conducted in accordance with its guidelines and the relevant national regulations.

## Supporting information


Appendix S1.

## Data Availability

The datasets analyzed during the current study are available from the corresponding author on reasonable request.
