# Supplementary material for: Repeated heat exposure upregulates skeletal muscle aquaporin‐4 expression in male mice
Source: Physiol Rep. 2026 May 19;14(10):e70926. doi: 10.14814/phy2.70926 (PMC13423740; doi:10.14814/phy2.70926)
Supplement: Supplementary file 1 — Appendix S1. [file PHY2-14-e70926-s001.pptx]

## Slide 1
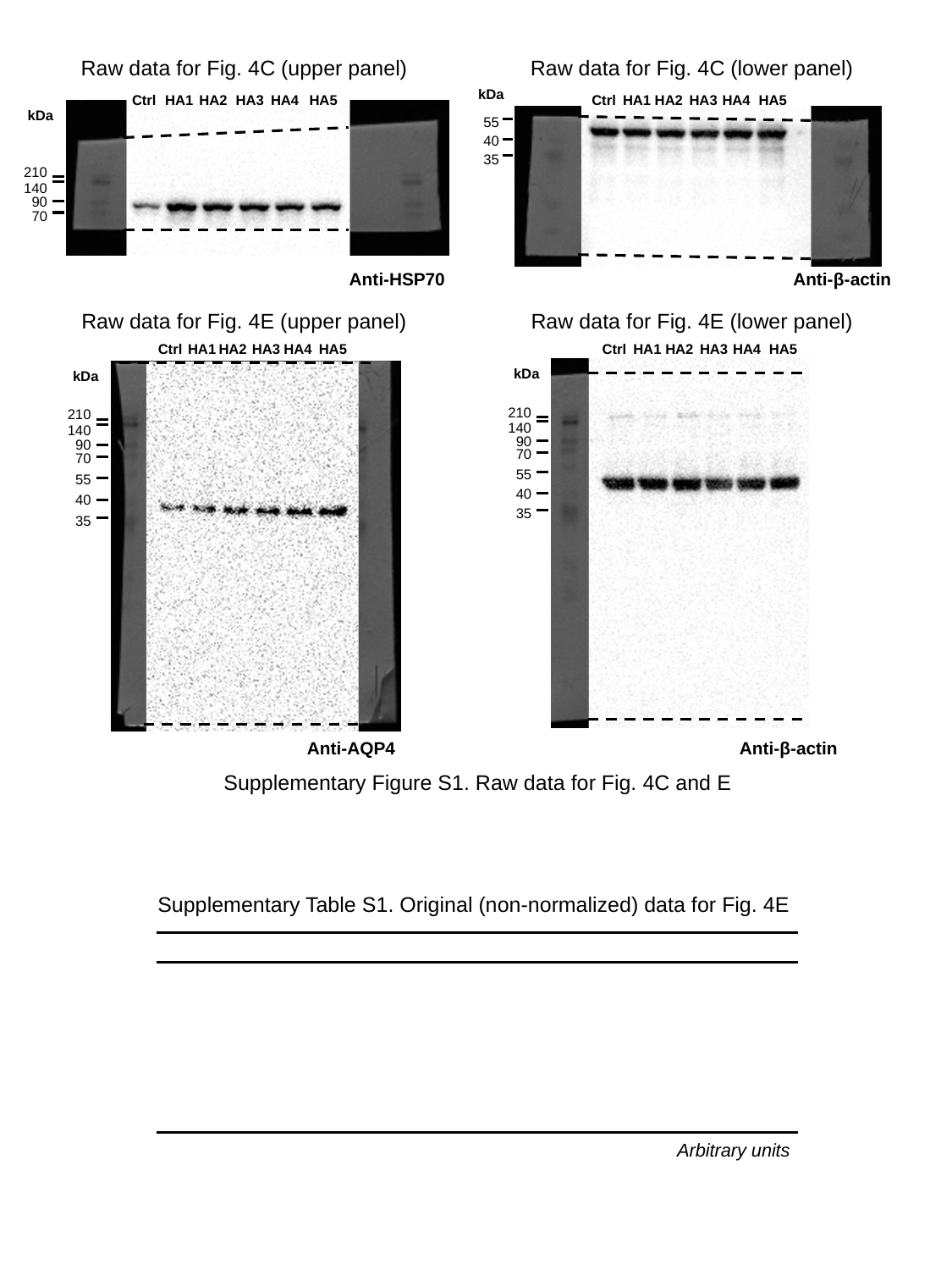

Raw data for Fig. 4C (upper panel)
Raw data for Fig. 4C (lower panel)
kDa
Ctrl
HA1
HA2
HA3
HA4
HA5
Ctrl
HA1
HA2
HA3
HA4
HA5
kDa
55
40
35
210
140
90
70
Anti-HSP70
Anti-β-actin
Raw data for Fig. 4E (upper panel)
Raw data for Fig. 4E (lower panel)
Ctrl
HA1
HA2
HA3
HA4
HA5
kDa
210
140
90
70
55
40
35
Anti-AQP4
Ctrl
HA1
HA2
HA3
HA4
HA5
kDa
210
140
90
70
55
40
35
Anti-β-actin
Supplementary Figure S1. Raw data for Fig. 4C and E
Supplementary Table S1. Original (non-normalized) data for Fig. 4E
Arbitrary units
